# Supplementary material for: Nondestructive detection of photonic qubits
Source: Nature. 2021 Mar 24;591(7851):570–4. doi: 10.1038/s41586-021-03290-z (PMC7990738; doi:10.1038/s41586-021-03290-z)
Supplement: Supplementary file 1 — This file contains supplementary text regarding the theory model for the NPQD and distillation of photonic Fock state qubits. [file 41586_2021_3290_MOESM1_ESM.pdf]

nature portfolio

https://doi.org/10.1038/s41586-021-03290-z

Supplementary information

Nondestructive detection of photonic qubits

In the format provided by the authors and unedited

Supplementary Information: Nondestructive detection of photonic qubits

D. Niemietz[1], P. Farrera[1,2], S. Langenfeld[1], and G. Rempe[1]

[1]Max-Planck-Institut für Quantenoptik, Hans-Kopfermann-Str. 1, 85748 Garching, Germany

[2]ICFO-Institut de Ciencies Fotoniques, The Barcelona Institute of Science and Technology, 08860 Castelldefels (Barcelona), Spain

January 20, 2021

1 Theory model for the NPQD

The measured parameters that characterize the nondestructive photonic qubit detector (NPQD) are shown in Fig. 3 in the main text together with numerical simulations. The theoretical model we employ is based on the cavity input-output theory [1] used in a similar way as in [2]. The simulations are written in python together with the QuTiP package [3].

We consider weak coherent pulses in front of the qubit cavity $|\alpha\rangle$ that are interacting with the atom-cavity system. After interaction, the photonic part might populate five different modes: the reflection at the resonator back into the fibre mode $r_{0_a,1_a}$ and back into the fibre cladding due to an imperfect fibre-cavity mode matching $r^o_{0_a,1_a}$, the transmission through the resonator $t_{0_a,1_a}$, and the scattering and absorption losses given by the fibre-cavity mirrors $m_{0_a,1_a}$ as well as by the atom $a_{0_a,1_a}$. The amplitudes of the arising weak coherent fields in the just mentioned modes are given

by:

$$
\begin{aligned}
r_{0_\mathrm{a},1_\mathrm{a}} &= \left(1-\mu_{FC}^2\frac{2\kappa_r}{Ng^2/(i\Delta_a+\gamma)+i\Delta_c+\kappa}\right)\alpha,\\
r^o_{0_\mathrm{a},1_\mathrm{a}} &= \sqrt{1-\mu_{FC}^2}\mu_{FC}\frac{2\kappa_r}{Ng^2/(i\Delta_a+\gamma)+i\Delta_c+\kappa}\alpha,\\
t_{0_\mathrm{a},1_\mathrm{a}} &= \mu_{FC}\frac{2\sqrt{\kappa_r\kappa_t}}{Ng^2/(i\Delta_a+\gamma)+i\Delta_c+\kappa}\alpha,\\
m_{0_\mathrm{a},1_\mathrm{a}} &= \mu_{FC}\frac{2\sqrt{\kappa_r\kappa_m}}{Ng^2/(i\Delta_a+\gamma)+i\Delta_c+\kappa}\alpha,\\
a_{0_\mathrm{a},1_\mathrm{a}} &= \mu_{FC}\frac{2g\sqrt{\kappa_r\gamma N}/(i\Delta_a+\gamma)}{Ng^2/(i\Delta_a+\gamma)+i\Delta_c+\kappa}\alpha.
\end{aligned}
\tag{1}
$$

Here, N is the number of atoms in the state that is coupled to the cavity mode (e.g. $N=0$ when the single atom is in state $|1_\mathrm{a}\rangle$ and $N=1$ when it is in $|0_\mathrm{a}\rangle$). The atom has an atomic dipole decay rate γ and a coupling strength with the qubit cavity mode of g. The spectral detuning between the incoming weak coherent field and the atomic transition (qubit cavity mode) frequency is given by Δ_a (Δ_c). κ_r, κ_t and κ_m describe the field decay rates into the respective mode with a total field decay rate of κ. Additionally, we take into account the imperfect fibre-cavity mode matching given by μ_{FC}. For convenience we simplify the notation of the state of the loss modes to $|l_{0_\mathrm{a},1_\mathrm{a}}\rangle :=$ $\left|r^o_{0_\mathrm{a},1_\mathrm{a}}, t_{0_\mathrm{a},1_\mathrm{a}}, m_{0_\mathrm{a},1_\mathrm{a}}, a_{0_\mathrm{a},1_\mathrm{a}}\right\rangle$. After the coherent pulse has interacted with the atom-cavity system, we can write the atom-photon state as

$$
\left|\Psi^1_{rla}\right\rangle = \frac{|r_{0_\mathrm{a}}, l_{0_\mathrm{a}}, 0_\mathrm{a}\rangle + |r_{1_\mathrm{a}}, l_{1_\mathrm{a}}, 1_\mathrm{a}\rangle}{\sqrt{2}}. \tag{2}
$$

A $\pi/2$ rotation operator is subsequently applied to the atomic part as the microwave pulse does in the experiment, leading to $|\Psi^2_{rla}\rangle = \mathbb{1}_r \otimes \mathbb{1}_l \otimes \hat{R}_a(\pi/2)\,|\Psi^1_{rla}\rangle$. With $|\Psi_{rla}\rangle$ we calculate the parameters that characterize our NPQD which is described in the following paragraphs.

Unconditional nondestructive detection probability $P(0_\mathrm{a})$: To investigate the detection probability $P(0_\mathrm{a})$, we first trace out the photonic part in order to obtain the atomic part ρ_a. Then, we

calculate the overlap with the photon detecting atomic state $|0_\mathrm{a}\rangle$:

$$\begin{aligned} \rho_a &= \mathrm{Tr}_{rl} \left|\Psi^2_{rla}\right\rangle \left\langle\Psi^2_{rla}\right| , \\ p_0 &= \langle 0_\mathrm{a}| \, \rho_a \, |0_\mathrm{a}\rangle , \end{aligned} \tag{3}$$

which provides the probability to nondestructively detect the input qubit photon. Due to NPQD dark counts, there is the small probability p_{DC} to detect the atom in state $|0_\mathrm{a}\rangle$ even if the NPQD did not interact with any photon. This is taken into account in order to obtain the final nondestructive detection probability

$$P(0_\mathrm{a}) = p_0 + (1 - p_0) \cdot p_{\mathrm{DC}}. \tag{4}$$

For an incoming weak coherent pulse with $|\alpha|^2 \to 0$, we expect p_0 to converge towards zero. However, eq. 4 shows that $P(0_a)$ is then governed by p_{DC} which sets the lower limit.

Nondestructive detection probability conditioned on qubit survival $P(0_\mathrm{a}| \geq 1_{\mathrm{oq}})$: This quantity is similarly evaluated as $P(0_\mathrm{a})$ but additionally underlies the condition of a reflected photonic qubit. In the simulation we therefor project the reflection mode of state $|\Psi^1_{rla}\rangle$ onto all non-vacuum components, using the projector operator $\hat{P}_r = \sum_{n_r>0} |n_r\rangle \langle n_r| \otimes \mathbb{1}_l \otimes \mathbb{1}_a$. The atom-photon state after this projection becomes $\rho^1_{la} = \hat{P}_r \rho^1_{rla} \hat{P}_r / \mathrm{Tr}(\hat{P}_r \rho^1_{rla} \hat{P}_r)$. In order to obtain the final atomic state, we consider the second atomic rotation $\rho^2_{la} = (\mathbb{1}_l \otimes \hat{R}_a(\pi/2)) \rho^1_{la} (\mathbb{1}_l \otimes \hat{R}_a(\pi/2))^\dagger$ and trace out the state of the photonic loss modes $\rho^2_a = \mathrm{Tr}_l(\rho^2_{la})$. Additionally, we add the density matrix that was projected onto the photonic vacuum state and weight it with the probability of measuring a conventional detector dark count. We finally calculate the overlap between the resulting density matrix and state $|0_\mathrm{a}\rangle$.

Mean photon number at the NPQD output conditioned on a nondestructive detection $\bar{n}_{\mathrm{oq}}(0_\mathrm{a})$: To compute $\bar{n}_{\mathrm{oq}}(0_\mathrm{a})$, the atomic part of state $|\Psi^2_{rla}\rangle$ is projected onto $|0_\mathrm{a}\rangle$. To end up with the state in reflection mode $\rho_r(0_\mathrm{a})$, the state of the photonic loss modes $|l_{0_\mathrm{a}}\rangle$ is traced out. With this, we get $\bar{n}_{\mathrm{oq}}(0_\mathrm{a}) = \langle \hat{n}_r \rangle_{\rho_r(0_\mathrm{a})}$ where $\hat{n}_r$ is the photon number operator acting on the state in reflection mode. Furthermore, we include the contribution of false nondestructive detection events due to

NPQD dark counts p_{DC} by

$$\tilde{n}_{\mathrm{oq}}(0_{\mathrm{a}}) = \frac{\bar{n}_{\mathrm{oq}}(0_{\mathrm{a}}) \cdot p_0 + \bar{n}_{\mathrm{oq}}(1_{\mathrm{a}}) \cdot (1 - p_0) \cdot p_{\mathrm{DC}}}{P(0_{\mathrm{a}})}, \tag{5}$$

where $\tilde{n}_{\mathrm{oq}}(0_{\mathrm{a}})$ is governed by p_{DC} as soon as p_0 converges towards zero whereas an absent p_{DC} would lead to the convergence towards $\bar{n}_{\mathrm{oq}}(0_{\mathrm{a}})$.

Autocorrelation function $g^{(2)}(0)$ of the NPQD output field upon nondestructive detection: Having $\rho_r(0_{\mathrm{a}})$ derived, it is an easy task to calculate the well known second order correlation function,

$$\tilde{g}^{(2)}(0) = \frac{\left\langle \hat{a}_r^\dagger \hat{a}_r^\dagger \hat{a}_r \hat{a}_r \right\rangle_{\rho_r(0_{\mathrm{a}})}}{\left\langle \hat{a}_r^\dagger \hat{a}_r \right\rangle^2_{\rho_r(0_{\mathrm{a}})}}, \tag{6}$$

where $\hat{a}_r$ is the photonic annihilation operator acting on the state in the reflection mode. In the limit of few detections for small $|\alpha|^2$, the dark counts of the SPDs result in an uncorrelated signal. Therefor, we include the conventional detector dark counts by

$$g^{(2)}(0) = \frac{g^{(2)}(0) \cdot p_{\geq 2} + 2 \cdot p_{\geq 1} \cdot p_{\mathrm{spd}} + p_{\mathrm{spd}}^2}{p_{\geq 2} + 2 \cdot p_{\geq 1} \cdot p_{\mathrm{spd}} + p_{\mathrm{spd}}^2}, \tag{7}$$

where $p_{\geq x}$ gives the integrated probability to find $\geq x$ photons in $\rho_r(0_{\mathrm{a}})$. p_{spd} describes the probability to measure a SPD dark count event.

2 Distillation of photonic Fock state qubits

An incoming single qubit photon would always imprint a π phase shift on the phase of the atomic superposition state of our NPQD. However, we have characterized the NPQD with weak coherent pulses that are composed of odd and even photon number contributions. This situation causes an ambiguous phase shift that is described by $n \cdot \pi$ with n as the photon number. Since the NPQD signal heralds a π phase shift, we conduct a projective parity measurement on the photon number state [2]. Moreover, in the range of $|\alpha|^2 \ll 1$ the nondestructive detection of an incoming qubit pulse leads to the distillation of an outgoing single photon that shows sub-Poissonian photon

statistics, $g^{(2)}(0) < 1$.

In the main text we provide a measurement of the second order correlation function of the reflected and nondestructively detected photonic pulses versus the mean photon number of incoming pulses. For this we have used the detection setup that consists of retardation waveplates, a polarizing beamsplitter and two SPDs (main text, Fig. 1b), enabling the realization of a Hanbury Brown-Twiss setup [4].

As already mentioned, we observe $g^{(2)}(0) < 1$ for $|\alpha|^2 \lessapprox 1$ (main text, Fig. 3b). However, in the limit of $|\alpha|^2 \to 0$, the dark counts of the SPDs result in an uncorrelated signal that ultimately limits the lowest value obtained for $g^{(2)}(0)$. On the other side, $|\alpha|^2 > 1$, we have first expected a convergence for $g^{(2)}(0)$ towards one. However, unequal field reflection coefficients (r_0 and r_1), as we have discussed them in Methods, lead to a state after reflection $\propto |r_0\alpha\rangle + |r_1\alpha\rangle$ which allows for photon statistics with $g^{(2)}(0) > 1$.

References

1. Kuhn, A. in *Engineering the Atom-Photon Interaction, Nano-Optics and Nanophotonics* (eds Predojević, A. & Mitchell, M. W.) 3–38 (Springer, Cham, 2015).

2. Daiss, S., Welte, S., Hacker, B., Li, L. & Rempe, G. Single-Photon Distillation via a Photonic Parity Measurement Using Cavity QED. *Phys. Rev. Lett.* **122,** 133603 (2019).

3. Johansson, J. R., Nation, P. D. & Nori, F. QuTiP: An open-source Python framework for the dynamics of open quantum systems. *Comput. Phys. Commun.* **183,** 1760–1772 (2012).

4. Hanbury Brown, R. & Twiss, R. Correlation between Photons in two Coherent Beams of Light. *Nature* **177,** 27 (1956).
